# Ligation of free HMGB1 to TLR2 in the absence of ligand is negatively regulated by the C-terminal tail domain

**DOI:** 10.1186/s10020-018-0021-x

**Published:** 2018-05-04

**Authors:** Hannah Aucott, Agnieszka Sowinska, Helena Erlandsson Harris, Peter Lundback

**Affiliations:** 1 Department of Medicine, Rheumatology Unit, Karolinska Institutet, Stockholm, Sweden; 2 GE Healthcare Life Sciences, Uppsala, Sweden; 3 Department of Medicine, Rheumatology Unit, Centre for Molecular Medicine (CMM) L8:04, Karolinska Hospital, 17176 Solna, Sweden

**Keywords:** HMGB1, TLR2, Alarmin, Receptor, Protein-protein interactions, Inflammation

## Abstract

**Background:**

High mobility group box 1 (HMGB1) protein is a central endogenous inflammatory mediator contributing to the pathogenesis of several inflammatory disorders. HMGB1 interacts with toll-like receptors (TLRs) but contradictory evidence regarding its identity as a TLR2 ligand persists. The aim of this study was to investigate if highly purified HMGB1 interacts with TLR2 and if so, to determine the functional outcome.

**Methods:**

Full length or C-terminal truncated (Δ30) HMGB1 was purified from *E.coli*. Binding to TLR2-Fc was investigated by direct-ELISA. For the functional studies, proteins alone or in complex with peptidoglycan (PGN) were added to human embryonic kidney (HEK) cells transfected with functional TLR2, TLR 1/2 or TLR 2/6 dimers, macrophages, whole blood or peripheral blood mononuclear cells (PBMCs). Cytokine levels were determined by ELISA.

**Results:**

In vitro binding experiments revealed that Δ30 HMGB1, lacking the acidic tail domain, but not full length HMGB1 binds dose dependently to TLR2. Control experiments confirmed that the interaction was specific to TLR2 and could be inhibited by enzymatic digestion. Δ30 HMGB1 alone was unable to induce cytokine production via TLR2. However, full length HMGB1 and Δ30 HMGB1 formed complexes with PGN, a known TLR2 ligand, and synergistically potentiated the inflammatory response in PBMCs.

**Conclusions:**

We have demonstrated that TLR2 is a receptor for HMGB1 and this binding is negatively regulated by the C-terminal tail. HMGB1 did not induce functional activation of TLR2 while both full length HMGB1 and Δ30 HMGB1 potentiated the inflammatory activities of the TLR2 ligand PGN. We hypothesize that Δ30 HMGB1 generated in vivo by enzymatic cleavage could act as an enhancer of TLR2-mediated inflammatory activities.

**Electronic supplementary material:**

The online version of this article (10.1186/s10020-018-0021-x) contains supplementary material, which is available to authorized users.

## Background

High mobility group box 1 (HMGB1), originally described as a nuclear DNA-binding protein, was re-discovered as an endogenous inflammatory mediator released from dying and activated cells in response to infectious and sterile stimuli in the late 90s (Wang et al. 1999). HMGB1 has since been identified as a pathogenic mediator during sepsis, arthritis, cancer, drug-induced liver injury and stroke, among other diseases, and inhibition of HMGB1 is beneficial in several experimental models (Andersson and Tracey 2011).

Structurally, HMGB1 expresses 215 amino acids organized into two DNA binding domains (boxes A and B) and a 30 amino acid long unstructured C-terminal acidic tail comprised of repeating glutamic and aspartic acid residues. Boxes A and B have a low sequence similarity (29%) but share a conserved global fold, consisting of three α-helices arranged in an L-like shape (Hardman et al. 1995; Weir et al. 1993). Biophyscial studies have shown that the tail interacts with residues in both boxes and the linker regions (Stott et al. 2010; Watson et al. 2007; Knapp et al. 2004). The interactions are dynamic with the protein alternating between “tail-bound” and “tail-unbound” conformations (Stott et al. 2010). Using circular dichroism spectroscopy Knapp et al. found that binding of the tail to specific residues within boxes A and B results in overall HMGB1 stabilization (Knapp et al. 2004). Moreover, binding of the tail to the boxes has been reported to modulate the interaction with other molecules including DNA and to regulate post-translational modifications (Lee and Thomas 2000; Muller et al. 2001; Sheflin et al. 1993; Stros et al. 1994; Pasheva et al. 2004).

Extracellular HMGB1 acts as an alarmin that interacts with multiple unrelated receptors to recruit and activate immune cells to the site of tissue damage. The receptor for advanced glycation end-products (RAGE), the toll-like receptor (TLR) family, C-X-C chemokine receptor 4 (CXCR4), cluster of differentiation 24 (CD24)/Siglec 10, TIM3 and integrin/Mac1 have all been described as HMGB1 receptors (Hori et al. 1995; Schiraldi et al. 2012; Das et al. 2016; Park et al. 2006; Chen et al. 2009; Gao et al. 2011; Chiba et al. 2012). Several members of the TLR family have been reported to interact with HMGB1 including TLR2, 4, 5 and 9 (Das et al. 2016; Yang et al. 2010; Park et al. 2004). Recent studies have defined HMGB1 binding to TLR4 and the subsequent NF-κB mediated cytokine production to be strictly regulated by post-translational cysteine redox modifications. Disulfide HMGB1 (dsHMGB1), containing a disulfide bridge between the two cysteine residues at positions 23 and 45 in box A and a reduced cysteine residue at position 106, binds to the TLR4/MD2 complex leading to NF-κB activation and expression of pro-inflammatory cytokines, including TNF (Yang et al. 2012; Yang et al. 2015). Complete reduction or oxidation of the three cysteine residues, generating fully reduced (frHMGB1) and sulfonyl (oxHMGB1) HMGB1 respectively, abolishes HMGB1-TLR4 mediated cytokine production (Yang et al. 2012). Likewise, mutation of the three cysteine residues to serines similarly abrogates HMGB1-TLR4 interactions (Venereau et al. 2012).

TLR2 and TLR4 were both suggested to be receptors for HMGB1 in a study by Park et al. in 2004 (Park et al. 2004) and since, the TLR2-HMGB1 axis has been suggested to contribute to the pathogenesis of several conditions including myocardial ischemia/reperfusion injury, peripheral artery disease, deep venous thrombosis and lupus nephritis (Stark et al. 2016; Feng et al. 2016; Mersmann et al. 2013; Sachdev et al. 2012; Xu et al. 2015). However, as compared to TLR4, much less is known about the mechanisms regulating the TLR2-HMGB1 interaction. TLR2 is found as a homodimer or a heterodimer in complex with TLR1 or 6 on the surface of a wide variety of cells including monocytes, tissue resident macrophages, T cells, B cells, dendritic cells and epithelial cells. Ligation of TLR2 activates MyD88-mediated signalling pathways increasing expression of pro-inflammatory genes. Interestingly, in vitro analysis of the interaction between HMGB1 and TLR2 has produced conflicting data. Early studies using fluorescence resonance energy transfer (FRET) and co-immunoprecipitation experiments suggested that HMGB1 binds directly to TLR2 on the surface of macrophages inducing NF-κB activation (Park et al. 2006). In two separate studies, TLR2 was reported to mediate HMGB1-induced cytokine production in human embroynic kidney (HEK) cells overexpressing TLR2 (Yu et al. 2006) and cancer stem cells (Conti et al. 2013). However, in 2010 Yang et al. concluded that TLR2 is not a major receptor mediating HMGB1-induced cytokine production (Yang et al. 2010). They showed that TLR2 or RAGE deficiency did not reduce HMGB1-induced secretion of TNF, MIP-2, IL-6, IL-8 and IL-10, in contrast to TLR4 deficiency which completely abrogated HMGB1-dependent cytokine expression (Yang et al. 2010).

To clarify conflicting data in the literature, the aim of this study was to investigate if highly purified HMGB1 interacts with TLR2 and if so, to determine the functional outcome of the interaction. Our results reveal that binding of HMGB1 to TLR2 is negatively regulated by the acidic C-terminal tail domain, with truncated HMGB1 lacking the tail domain displaying dose-dependent binding to TLR2-Fc in contrast to full length HMGB1 (flHMGB1). We could not detect any functional consequence of the direct binding interaction between Δ30 HMGB1 and TLR2 in vitro. However, we can demonstrate that Δ30 HMGB1 forms complexes with known TLR2 ligands that enhance downstream signaling and cytokine production.

## Methods

### Preparation of recombinant HMGB1 proteins

**Fig. 1:**
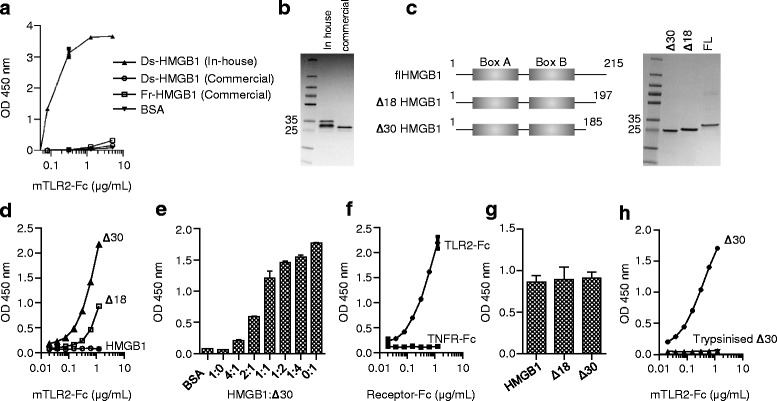
The C-terminal acidic tail domain inhibits binding of HMGB1 to TLR2. **a**) Binding of HMGB1 to TLR2 was investigated by ELISA. Plates were coated with different batches of HMGB1 and incubated with increasing concentrations of TLR2-Fc. No interaction between commercial HMGB1 and TLR2-Fc was detected. In contrast, in-house produced HMGB1 bound to TLR2-Fc in a dose-dependent manner. Ds-HMGB1 = disulfide HMGB1, Fr-HMGB1 = fully reduced HMGB1. **b**) SDS-page gel electrophoresis analysis of the in-house and commercial proteins confirmed that the commercial preparation only contained full length HMGB1 whilst the in-house preparation was a mixture of full length and C-terminus truncated protein. **c**) Schematic structure and SDS-page gel analysis of full length and C-terminal truncated HMGB1 proteins (Δ18 and Δ30) **d**) Δ30 and Δ18 with a full or partially truncated C-terminus bind to TLR2-Fc as detected by ELISA **e**) Increasing concentrations of Δ30 results in increasing binding to TLR2-Fc. **f**-**h**) Control experiments to confirm that the interaction is specific to TLR2 (**f**), is not due to differences in coating of the recombinant proteins to the ELISA plates (**g**) and can be inhibited using enzymatic digestion of the Δ30 protein (**h**). Representative data shown from 3 to 5 experiments. In **e**, **f** and **g** BSA is represented by an opened triangle

For the experiment depicted in Fig. 1, in-house HMGB1 with a CBP tag was expressed and purified as previously described (Yang et al. 2010). Briefly, HMGB1 DNA was sub-cloned into the pCAL/n vector with a calmodulin binding protein (CBP) tag. The plasmid was transformed into *E.coli* BL21 (DE3) cells and cultured in 2-YT media. Protein expression was induced with the addition of 1 mM IPTG. HMGB1 was purified using calmodulin sepharose 4B resin (GE Healthcare). DNase I was added to remove any contaminating DNA, confirmed by GelRed staining of an agarose gel. Protein purity was verified by SDS-PAGE gel analysis with Coomassie blue staining. For the removal of contaminating endotoxin, the protein was incubated with 1% Triton X114 at 4 °C for 30 min, incubated for a further 10 min at 37 °C and centrifuged at 18,300 g at 25 °C for 10 min. Endotoxin levels were measured using the limulus amoebocyte lysate assay at the clinical laboratory, Karolinska University Hospital, Stockholm, Sweden.

Commercial ds- and fr-HMGB1 were purchased from HMG Biotech (Milan, Italy).

Tag free human flHMGB1 and Δ30 HMGB1 (lacking the C-terminal tail; residues 1–185) were cloned into the pETM-11 vector and expressed in *Escherichia coli* strain BL21 (DE3) cells. The proteins expressed a 6-residue N-terminal histidine tag (his-tag) with a tobacco etch virus (TEV) cleavable linker and were purified using Ni-sepharose affinity chromatography (HisTrap HP column, GE Healthcare, Uppsala, Sweden) on an ÄKTA explorer (GE Healthcare). A partially truncated HMGB1 (∆18 HMGB1) was generated as a by-product during the production of the flHMGB1 protein and was co-purified during the affinity purification. Binding of the N-terminal his-tag to the column confirmed that the truncation was at the C-terminal tail domain. Truncated HMGB1 and flHMGB1 were separated using anion exchange chromatography on a HiTrap Q FF column (GE Healthcare) using an increasing salt concentration. The his-tag was cleaved using TEV protease (Protein science facility, Karolinska Institutet, Sweden) at a ratio of 1:20. Endotoxin was removed using Triton-X114 two phase extraction (Aida and Pabst 1990); all proteins preparations had endotoxin levels below 0.03 EU/μg as measured using the limulus amebocyte lysate (LAL) assay (Department of Clinical Microbiology, Karolinska Universitetssjukhuset). Protein purity was confirmed using SDS-PAGE gel electrophoresis analysis.

### TLR2 binding assay

96 well microtiter plates were coated with 40 nmol of protein (HMGB1, Δ18 HMGB1, Δ30 HMGB1) in PBS, pH 7.2 for 2 h at 37 °C. Plates were washed (0.05% Tween 20 in PBS, pH 7.2 was used as the buffer for all washing steps) and blocked with 1% bovine serum albumin (BSA) for 1 h. After washing, 0.2–1.25 μg/mL TLR2-Fc (R&D systems, UK) diluted in 1% BSA in PBS, pH 7.2 was added and plates were incubated for a further 2 h. For detection of bound TLR2-Fc, plates were first incubated with anti-human IgG HRP diluted in 1% BSA in PBS, pH 7.2 (1/1000; Dako, Agilent Technologies, Stockholm, Sweden) for 1 h and then 3, 3′, 5, 5′-Tetramethylbenzidine *(*TMB*)* substrate solution (Sigma Aldrich, Stockholm, Sweden). The reaction was stopped by adding 2 N H_2_SO_4_. In control experiments, 0.2–1.25 μg/mL Etanercept (TNFR-Fc; Pfizer, Sandwich, UK) or 80 ng/mL h2G7 antibody (humanized anti-HMGB1 (Lundback et al. 2016); the binding epitope for 2G7 is between residues 53 and 63 in HMGB1) was added in place of TLR2-Fc.

Enzymatic digestion of Δ30 HMGB1 was performed on column using immobilized trypsin according to the manufacturer’s instructions (Promega, Madison, WI, USA). Briefly, the protein was denatured at 85 °C for 5 min, diluted in digestion buffer (50 mM ammonium bicarbonate, 40% ACN) and incubated on the column overnight at room temperature. Peptides were eluted in the recovery buffer (50 mM ammonium bicarbonate, 40% ACN, 0.02% TCA) by centrifugation and coated to ELISA plates as described previously.

### HEK293 and alveolar macrophage (MH-S) cell culture conditions

HEK293 cells stably transfected with functional TLR2, TLR1/2 and TLR2/6 dimers (Invivogen, Toulouse, France) were cultured in DMEM (Sigma Aldrich) supplemented with heat inactivated fetal bovine serum (FBS) (10%; Sigma Aldrich), penicillin (100 U/mL)-streptomycin (100 μg/mL; Sigma Aldrich), L-glutamine (2 mM; Sigma Aldrich), normocin (100 μg/mL; Invivogen) and blasticidin (10 μg/mL; Invivogen) at 37 °C with 5% CO_2_.

The alveolar macrophage cell line, MH-S, (kindly provided by Fredrick Wermeling’s laboratory, Karolinska Institutet, Stockholm, Sweden) was cultured in RPMI media (Sigma Aldrich) supplemented with heat inactivated FBS (10%; Sigma Aldrich), penicillin (100 U/mL)-streptomycin (100 μg/mL; Sigma Aldrich) and 2-mercaptoethanol (0.05 mM; Sigma Aldrich) at 37 °C with 5% CO_2_.

For experiments, 0.05 × 10^4^ cells were seeded in 96 well microtiter plates in serum free media and left to rest for 6 h. Recombinant proteins (flHMGB1 or Δ30 HMGB1) or positive controls (TLR4 control: LPS (Sigma Aldrich) and TLR2 controls: peptidoglycan (PGN, 2.5 μg/mL) and Pam3CSK4 (2.5 μg/mL; Invivogen)) were diluted in PBS and added at indicated concentrations. Supernatants were collected 24 h after dosing and stored at -20 °C until analysis.

### Whole blood assay

Blood was collected from adult healthy donors into sodium-heparin tubes and added to a 96 well microtiter plate (180 μL/well; 200 μL final volume). Recombinant proteins (flHMGB1 or Δ30 HMGB1) or controls (LPS (100 ng/mL), PGN (2.5 μg/mL) and Pam3CSK4 (2.5 μg/mL)) were diluted in PBS and added at the concentrations indicated. Plates were incubated at 37 °C for 4 h. Samples were centrifuged at 2000 g for 10 min, the serum was collected and stored at -20 °C until analysis. The study was approved by the North Ethical Committee in Stockholm, Sweden.

### Peripheral blood mononuclear cells (PBMCs)

Blood was collected from adult healthy donors and PBMCs were purified using Ficoll centrifugation (Ficoll-Paque Plus, GE healthcare). Cells were seeded at 0.1 × 10^6^ cells/well in a 96 well plate in Opti-MEM (Invitrogen) and cultured at 37 °C with 5% CO_2_. Cells were treated with Δ30 HMGB1 or flHMGB1 (0–2000 ng/mL) alone or protein that had been pre-incubated with 20 ng/mL PGN for 16–18 h at 4 °C as 40 x stock solutions. The concentration of PGN was determined by performing a titration experiment and did not induce cytokine production alone. Supernatants were collected 24 h after protein addition. The study was approved by the Stockholm North Ethical Committee in Stockholm, Sweden.

### Elisa

Cytokine levels were determined by ELISA according to the manufacturer’s instructions (R&D systems, UK).

### Statistical analysis

Statistical analysis was performed using graphpad Prism v6 software. Data was analyzed using a Kruskal-Wallis test with Dunn’s multiple comparison correction. * *p* ≤ 0.05, ** *p* ≤ 0.01, *** *p* ≤ 0.001.

## Results

### The C-terminal tail inhibits the interaction with TLR2

To investigate the binding of HMGB1 to TLR2, we coated ELISA plates with different batches of HMGB1 purified in-house (CBP-HMGB1) or purchased commercially (tag-free HMGB1) and incubated with increasing concentrations of TLR2-Fc. Since a number of recent studies have revealed that HMGB1 binding to TLR4 is strictly regulated by cysteine redox modifications, we tested the binding of both fr- and ds-HMGB1 isoforms. As shown in Fig. 1a, the in-house produced CBP-HMGB1 displayed dose-dependent binding to TLR2-Fc, in contrast to the commercial tag-free HMGB1 which did not interact with TLR2; either in the reduced or disulfide isoform. From our previous experience, we knew that recombinant HMGB1 produced in-house with an N-terminal tag often comprises of a mixture of full length and C-terminally truncated proteins, in contrast to commercial preparations, which only contain the flHMGB1 protein. We confirmed that this was the case using SDS-PAGE gel electrophoresis (Fig. 1b) and hypothesized that the difference in the ability to bind to TLR2 might be explained by the presence of the C-terminal truncated protein in the in-house produced HMGB1 batch.

To test this hypothesis, we purified a new batch of tag-free HMGB1 and separated the full length and truncated HMGB1 proteins using ion exchange chromatography (Additional file 1: Figure S1A & B). Protein purity was confirmed using SDS-PAGE gel electrophoresis (Fig. 1c) and accurate molecular weight (MW) was determined for the partially truncated proteins using mass spectrometric analysis. The results confirmed that the molecular weight was between 22.5 and 23.2 kDa, consistent with the loss of 13 to 18 residues from the C-terminal tail domain (referred to as Δ18; Additional file 1: Figure S1C). In addition, we also expressed and purified a protein with a full deletion of the C-terminal tail domain (Δ30; Fig. 1c).

The ability of the different HMGB1 proteins to bind to TLR2-Fc was investigated using the binding assay. Δ30 HMGB1 dose-dependently bound to TLR2-Fc as did Δ18 HMGB1 although to a much lower extent (Fig. 1d). In contrast to Δ18 HMGB1 and Δ30 HMGB1, full length tag-free HMGB1 did not interact with TLR2-Fc confirming that the binding interaction is inhibited in the presence of the C-tail domain. Moreover, protein mixtures with increasing concentrations of truncated HMGB1 compared to the FL protein displayed higher binding to TLR2-Fc (Fig. 1e and Additional file 1: Figure S2).

To confirm that the binding was specific to TLR2 and not caused by non-specific interactions with the Fc domain, plates were coated with Δ30 HMGB1 and incubated with increasing concentrations of TLR2 or TNFR-Fc. As shown in Fig. 1f, Δ30 HMGB1 did not interact with TNFR-Fc confirming the specificity to TLR2. Additionally, Δ30 HMGB1 did not bind to an irrelevant human IgG1 isotype control antibody (data not shown). Furthermore, all three HMGB1 proteins displayed equivalent binding to h2G7, a partly humanized anti-HMGB1 monoclonal antibody (Lundback et al. 2016) thus confirming that the results observed were not caused by differences in the coating of the proteins to the ELISA plates (Fig. 1g). Finally, to demonstrate that binding to TLR2-Fc was specific to Δ30 HMGB1 and not due to any potential protein contaminates, the protein was enzymatically digested using trypsin prior to the ELISA experiment. Enzymatically digested Δ30 HMGB1 did not bind to TLR2-Fc (Fig. 1h).

### Pure Δ30 HMGB1 does not mediate TLR2-mediated cytokine production in vitro

**Fig. 2:**
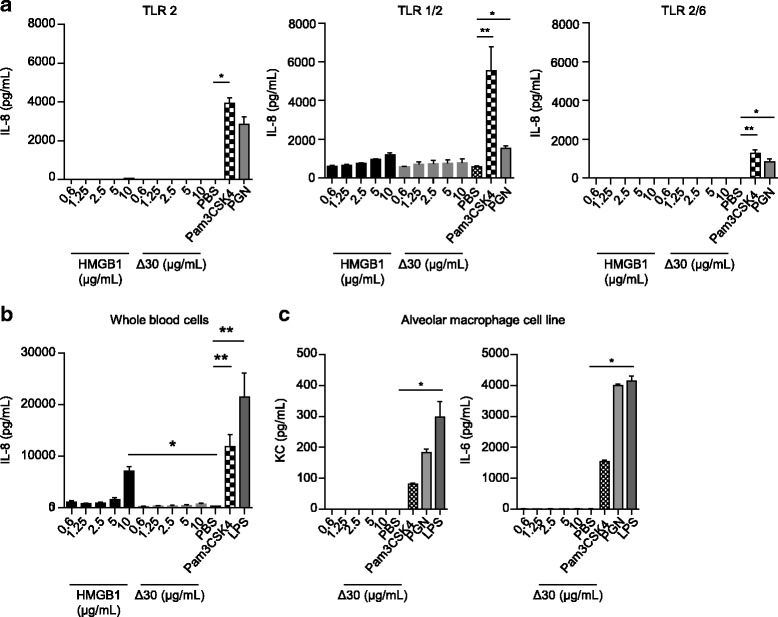
Pure C-tail truncated HMGB1 (Δ30) does not mediate cytokine production in vitro. HMGB1 and Δ30 were added at the indicated concentrations to **a**) HEK cells transfected with functional TLR 2, 1/2 and 2/6 dimers, **b**) whole blood collected from healthy volunteers and **c**) alveolar macrophage cells. Serum cytokine levels were quantified by ELISA after 24 h. PGN (2.5 μg/mL) and Pam3CSK4 (2.5 μg/mL), known TLR2 ligands, were included as positive controls. LPS (100 ng/mL) was included as a TLR4 positive control. (**a** = representative data from 1/3 experiments; **b** = combined data from 5 individual donors; **c** = 1 experiment performed in triplicate wells)

In vivo TLR2 has been reported to signal as a homodimer or as a heterodimer complexed with TLR1 or TLR6. To elucidate the functional relevance of the interaction discovered between Δ30 and TLR2 in the binding studies, HEK293 cells expressing functional TLR2, 1/2 or 2/6 dimers were incubated with Δ30 HMGB1 or flHMGB1 for 24 h and the supernatants were analyzed for cytokine production. Pam3CSK4 and PGN, known TLR2 ligands, were included as positive controls and induced IL-8 production in all of the cell lines. Δ30 HMGB1 and flHMGB1 did not induce cytokine production in the cells transfected with TLR2 or TLR2/6 (Fig. 2a). Moreover, Δ30 HMGB1 did not increase cytokine levels in the TLR1/2 transfected cells, compared to the PBS control. However, addition of flHMGB1 resulted in a non-significant dose-dependent increase in IL-8 production in the TLR1/2 cells.

To confirm that the absence of cytokine production in the TLR-transfected HEK293 cells was not due to the lack of additional co-factors that may be required for receptor binding and downstream signaling pathways, we isolated whole blood from healthy volunteers and incubated the cells with the recombinant HMGB1 proteins previously tested in the ELISA experiments. No cytokine production was detected in the PBS control or in the cells treated with Δ30 HMGB1 (Fig. 2b). flHMGB1, which did not interact with TLR2-Fc in the binding experiments (Fig. 1e), significantly increased IL-8 levels at 10 μg/mL but not at lower concentrations possibly by interacting with other receptors present on the cells including TLR4. Moreover, Δ30 HMGB1 did not induce cytokine production in the alveolar macrophage cell line expressing high levels of CD36, a co-receptor for TLR2 heterodimer signaling (Hoebe et al. 2005) (Fig. 2c).

### HMGB1 acts in synergy with other molecules to enhance TLR2-mediated cytokine production

**Fig. 3:**
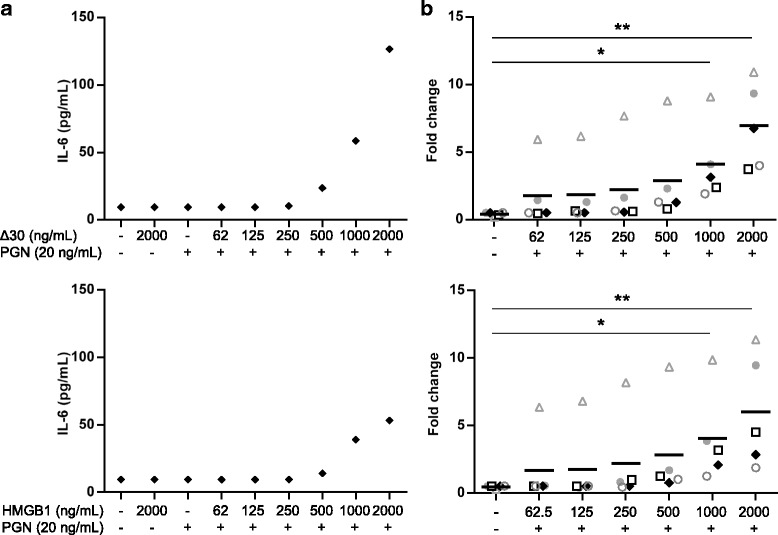
HMGB1 enhances TLR2 responses to peptidoglycan. PBMCs were isolated from healthy controls and stimulated with HMGB1 or Δ30 alone or in complex with peptidoglycan (PGN) at the indicated concentrations. Supernatants were collected after 24 h and IL-6 levels were measured by ELISA. **a**) Representative cytokine levels from 1 healthy control and **b**) Fold change in IL-6 level compared to the control group (IL-6 release with HMGB1 alone + IL-6 release with PGN alone). *n* = 5 donors

In addition to acting directly as a potent inflammatory mediator, it has previously been demonstrated that HMGB1 synergistically enhances the response to several endogenous and exogenous molecules including LPS, CXCL12 and IL-1β (Hreggvidsdottir et al. 2012; Hreggvidsdottir et al. 2009; Wahamaa et al. 2011; Schiraldi et al. 2012). Since we did not record any direct consequences of Δ30 HMGB1 binding to TLR2 we hypothesized that Δ30 HMGB1 may enhance TLR2-mediated responses. To test this, isolated PBMCs from healthy controls were incubated with Δ30 HMGB1 or flHMGB1 in complex with PGN. Addition of PBS, Δ30 HMGB1, flHMGB1 or PGN alone did not result in any detectable cytokine production (Fig. 3a). However, Δ30 HMGB1 and flHMGB1 in complex with PGN significantly enhanced IL-6 cytokine production by 7- and 6-fold respectively (Fig. 3a & b).

## Discussion

A detailed understanding of HMGB1 signaling pathways is required to fully elucidate how HMGB1 contributes to disease pathogenesis and to identify novel therapeutic targets. It is becoming increasingly clear that post-translational modifications generate several different and functional HMGB1 isoforms that have the ability to interact with multiple unrelated receptors. While recent studies have defined HMGB1 binding to TLR4, little is known about the molecular requirements for HMGB1 interactions with TLR2. Consequently, the aim of this study was to investigate if TLR2 is a functional receptor for HMGB1 and if so, to identify the molecular requirements for binding. The results confirm that HMGB1 directly interacts with TLR2, but this interaction is inhibited by the presence of the C-terminal tail domain (Fig. 1d). Although it is able to directly bind to TLR2, highly purified Δ30 HMGB1 per se does not induce cytokine production via this receptor (Fig. 2), however it does act synergistically with known TLR2 ligands to potentiate their effects (Fig. 3).

In the current study, we are able to show that the conflicting findings previously reported on the HMGB1-TLR2 interaction are likely explained by the use of different sources of recombinant HMGB1. In our, and others, experience the expression of recombinant HMGB1 containing an N-terminal tag for purification purposes often results in the production of both flHMGB1 and a C-terminal truncated protein (Fig. 1c & Additional file 1: Figure S1) (Li et al. 2004). The generation of C-terminal truncated HMGB1 is most likely caused 1) by rapid degradation of the newly synthesized protein due to toxic effects of the acidic tail (Bianchi 1991; Lee et al. 1998) or 2) an inability of the bacteria to effectively transcribe the repetitive C-tail domain. As shown here, only truncated HMGB1 can interact with TLR2 (Fig. 1d). However, even a partial depletion of the tail (13–18 residues) is sufficient for TLR2 binding to occur (Fig. 1d). Further studies are needed to identify whether the binding events are steered by electrostatic or hydrophobic forces and the critical residues required for the interaction.

The acidic tail regulates binding of HMGB1 to other molecules by interacting with the A and B boxes (Stott et al. 2010; Knapp et al. 2004). Interactions with box A are favoured and involve specific residues outside of the basic lysine-rich domains. Generally, depletion of the tail increases the affinity for molecules that bind to the boxes or linker residues. Removal of the tail increases the affinity for both linear and distored DNA structures and increases the DNA bending ability (Lee and Thomas 2000; Sheflin et al. 1993; Stros et al. 1994; Pasheva et al. 1998). In addition, acetylation of lysine 81 by CREB-binding protein (CBP) is inhibited when the C-terminus tail is present (Pasheva et al. 2004). The tail is required for binding to the H1 and H3 histones (Cato et al. 2008; Watson et al. 2013) and metformin (Horiuchi et al. 2017). Moreover, here we show that the tail inhibits binding of HMGB1 to TLR2 however, a recent study found that the tail is required for the interaction with TLR5 (Das et al. 2016) suggesting that the tail regulates HMGB1 ligation to different TLRs.

Redox modification of the three cysteine residues present in HMGB1 regulates the inflammatory function and binding to TLR4 (Yang et al. 2010; Yang et al. 2012; Venereau et al. 2012). From these experiments it is difficult to define how the redox status impacts on the interaction of HMGB1 with TLR2. However, we observed that whilst the addition of H_2_O_2_ did not alter binding of Δ30 to TLR2-Fc, exposure to DTT reduced the interaction (Additional file 1: Figure S3A), indictaing that redox modification may affect the binding. Treatment with H_2_O_2_ or DTT had no impact on the interaction between flHMGB1 and TLR2; in all cases flHMGB1 did not bind to TLR2 (Additional file 1: Figure S3B). This would suggest that although redox modification may contribute to the binding of HMGB1 to TLR2, it is the presence of the C-tail domain that is the major regulator of the interaction.

In additional to post-translational redox modifications, HMGB1 has been found to be a target for acetylation, phosphorylation, and methylation events (Tang et al. 2016). Acetylation of key lysine residues within the two nuclear localization signal (NLS) domains is required for cytoplasmic accumulation and active secretion of HMGB1 from monocytes and macrophages (Bonaldi et al. 2003). HMGB1 released from monocytes stimulated with LPS is also acetylated at several lysine residues found outside of the NLS sites (Lu et al. 2014). We speculate that acetylation of HMGB1 may alter the electrostatic potential and disrupt the interaction between the tail and the boxes possibly revealing the TLR2 binding sites in vivo*,* although this requires further investigation.

The interactions between the boxes and the tail may also be disrupted through the binding of a partner molecule to HMGB1. Previous studies have demonstrated that HMGB1 is able to interact with a wide variety of endogenous and exogenous molecules including Pam3CSK4, a synthetic TLR2 ligand (Hreggvidsdottir et al. 2009). HMGB1 complexes have enhanced pro-inflammatory activity and signal via the partner molecule receptor (Hreggvidsdottir et al. 2012). Here, we demonstrate that HMGB1 enhances PGN induced cytokine production via TLR2. HMGB1 also enhances PGN induced iNOS expression and nitric oxide release in macrophages (Chakraborty et al. 2013). Full length HMGB1 and Δ30 HMGB1 complexes with PGN induced comparable cytokine production (Fig. 3b), suggesting that the binding site(s) for PGN is located in the A box, B box or the linker residues. Binding of PGN to flHMGB1 may displace the interaction between the acidic tail and the boxes thereby exposing TLR2 binding epitopes.

HMGB1 complexes with LPS and CXCL12 mediate inflammation by enhancing cytokine production and inflammatory cell recruitment, respectively (Schiraldi et al. 2012; Hreggvidsdottir et al. 2009). We speculate that Δ30 HMGB1 may also bind to LPS and CXCL12 since the binding sites for both molecules have been mapped to boxes A and B, which are present in Δ30 HMGB1 and are likely to be exposed in the absence of the tail domain (Schiraldi et al. 2012; Youn et al. 2011). Δ30 HMGB1 also interacts with IL-1ß to enhance cytokine production in synovial fibroblasts isolated from arthritic patients (unpublished data from our lab).

Previous in vivo data suggests that the HMGB1-TLR2 axis may contribute to the pathogenesis of several inflammatory diseases, and that inhibition of this pathway may improve clinical outcome (Stark et al. 2016; Feng et al. 2016; Mersmann et al. 2013; Curtin et al. 2009). However, the results from the current study indicate that C-terminal truncated HMGB1 alone, containing no post-translational modification or binding partner, cannot signal via TLR2 to induce cytokine production (Fig. 2). Rather, our data suggests that HMGB1-TLR2 pro-inflammatory responses could be mediated by HMGB1-complexes (Fig. 3).

In the current study we have focused on investigating if HMGB1 induces TLR2-dependent cytokine production. However, recent studies also suggest a role for TLR2 in mediating protective and regenerative effects in models of skeletal muscle ischemia (Sachdev et al. 2012). Sachdev et al. found that TLR2 signalling reduces necrosis and promotes monocyte regeneration by attentuating HMGB1/TLR4 pathways (Xu et al. 2015). Moreover, TLR2 signalling is required for HMGB1-induced angiogenesis (Xu et al. 2015).

## Conclusions

In summary, we have demonstrated that the C-terminal tail of HMGB1 negatively regulates the interaction with TLR2. The results from this study together with other reports thus indicate that binding of HMGB1 to different TLRs is differentially regulated. Disulfide full length HMGB1 interacts with TLR4 but cannot bind to TLR2 (Fig. 1a), which requires partial or full loss of the C-terminal tail (Fig. 1d). Binding of truncated HMGB1 to TLR2 does not appear to induce cytokine production alone, rather it acts in synergy with other TLR2 ligands. Further studies are needed to determine if a truncated HMGB1 protein similar to Δ18 or Δ30 exists in vivo and can be isolated from biological fluids. A study by Sterner and colleagues found that a C-terminal truncated HMGB1 protein may be present in calf thymus chromatin (Sterner et al. 1979). We hypothesize that such a protein may be generated by proteolytic cleavage of HMGB1 and that this molecule could act as an enhancer of TLR2-mediated inflammatory activities.

## Additional file

Additional file 1: Supplementary information. (PDF 447 kb)

## Abbreviations

BSA: Bovine serum albumin
CBP: Calmodulin-binding peptide
CD24: Cluster of differentiation 24
CXCR4: C-X-C chemokine receptor 4
dsHMGB1: Disulfide HMGB1
DTT: Dithiothreitol
flHMGB1: Full length HMGB1
frHMGB1: Fully reduced HMGB1
H_2_O_2_: Hydrogen peroxide
HEK: Human embryonic kidney
HMGB1: High mobility group box 1
LPS: Lipopolysaccharide
NLS: Nuclear localization signal
oxHMGB1: Sulfonyl HMGB1
PBMC: Peripheral blood mononuclear cell
PGN: Peptidoglycan
RAGE: Receptor for advanced glycation end-products
TEV: Tobacco etch virus
TLR: Toll-like receptor
