# Supplementary material for: Ligation of free HMGB1 to TLR2 in the absence of ligand is negatively regulated by the C-terminal tail domain
Source: Mol Med. 2018 May 4;24:19. doi: 10.1186/s10020-018-0021-x (PMC6016865; doi:10.1186/s10020-018-0021-x)
Supplement: Supplementary file 1 — Supplementary information. (PDF 447 kb) [file 10020_2018_21_MOESM1_ESM.pdf]

Supplementary methods

Sample preparation

The protein solution was prepared using a Nanosep 10K Omega centrifugal device (Pall Life Sciences, Ann Arbor, MI) adding 27 µg protein diluted in 500 µL of 20 mM of ammonium acetate. Following wash with 500 µL of 20 mM of ammonium acetate twice, proteins were collected in 40 µL of buffer, resulting in ca. 24 µg (calculated at 90% recovery rate). Five µL of this protein solution was mixed with 5 µL of 5% formic acid and sprayed into the mass spectrometer.

Mass Spectrometry

Sample analysis was performed using a TriVersa NanoMate chip-based electrospray device (Advion, Ithaca, NY) coupled to the LTQ Velos Orbitrap Elite (Thermo Scientific, Bremen, Germany). The ChipSoft Manager software was used to control the TriVersa NanoMate, while data was acquired directly from the Tune software of the mass spectrometer. The NanoMate delivered 2 µL of sample solution to the tip engaged with the back of the ESI chip and nano-spray ionization was initiated applying 1.9 kV and 0.8 psi gas pressure. The mass spectrometer was operated in positive ion mode with activated protein mode settings. MS data was collected in full scan mode (*m/z* 500-2000) with a resolution of 100,000 at *m/z* 400. Each scan comprises 1 microscan. The mass spectra shown are comprised of approximately 20 scans. Automatic gain control (AGC) was used to accumulate sufficient ions for analysis targeting $3x10^7$ ions in a maximum fill time of 200 ms. Data were analyzed using Xcalibur 2.10 software (Thermo Scientific) where the Xtract program was used to calculate monoisotopic masses.

Supplementary figure legends

Fig S1. Production and characterization of tag-free full length and C-terminal truncated (Δ18) HMGB1 A) Recombinant HMGB1 was expressed in BL21 (DE3) cells and the lysate was purified using affinity chromatography. Two bands of different molecular weights (MW) were identified using SDS-PAGE gel electrophoresis. B) HMGB1 was further separated using ion exchange chromatography to obtain purified full length and C-terminal truncated proteins. C) Accurate MW analysis of the C-terminal truncated protein was performed using electrospray ionization mass spectrometry (ESI MS). The MW was consistent with the removal of between 13 to 18 residues from the C-terminus (Since the protein bound to the column via the N-terminal His-tag; Δ13-18; referred to as Δ18).

Fig S2. Partial or complete depletion of the C-terminal tail domain is required for HMGB1 binding to TLR2 A) Pure mixtures of full length and C-tail truncated HMGB1 proteins were prepared and analysed by SDS-page gel electrophoresis. B) Protein mixtures were coated onto ELISA plates and incubated with increasing concentrations of TLR2-Fc. The presence of the C-terminal reduced the binding of TLR2 to HMGB1.

Fig S3. Binding of HMGB1 and Δ30 to TLR2-Fc in the presence of DTT or H_2O_2 Binding of Δ30 (A) or HMGB1 (B) to TLR2-Fc was investigated by ELISA. Plates were coated onto microtiter plates in the presence of 5mM DTT or 10mM H_2O_2 and incubated with increasing concentrations of TLR2-Fc. H_2O_2 did not impact the binding of Δ30 to TLR2-Fc, however DTT reduced the interaction. HMGB1 did not interact with TLR2-Fc in any of the conditions tested (n = 2)

Fig S1.

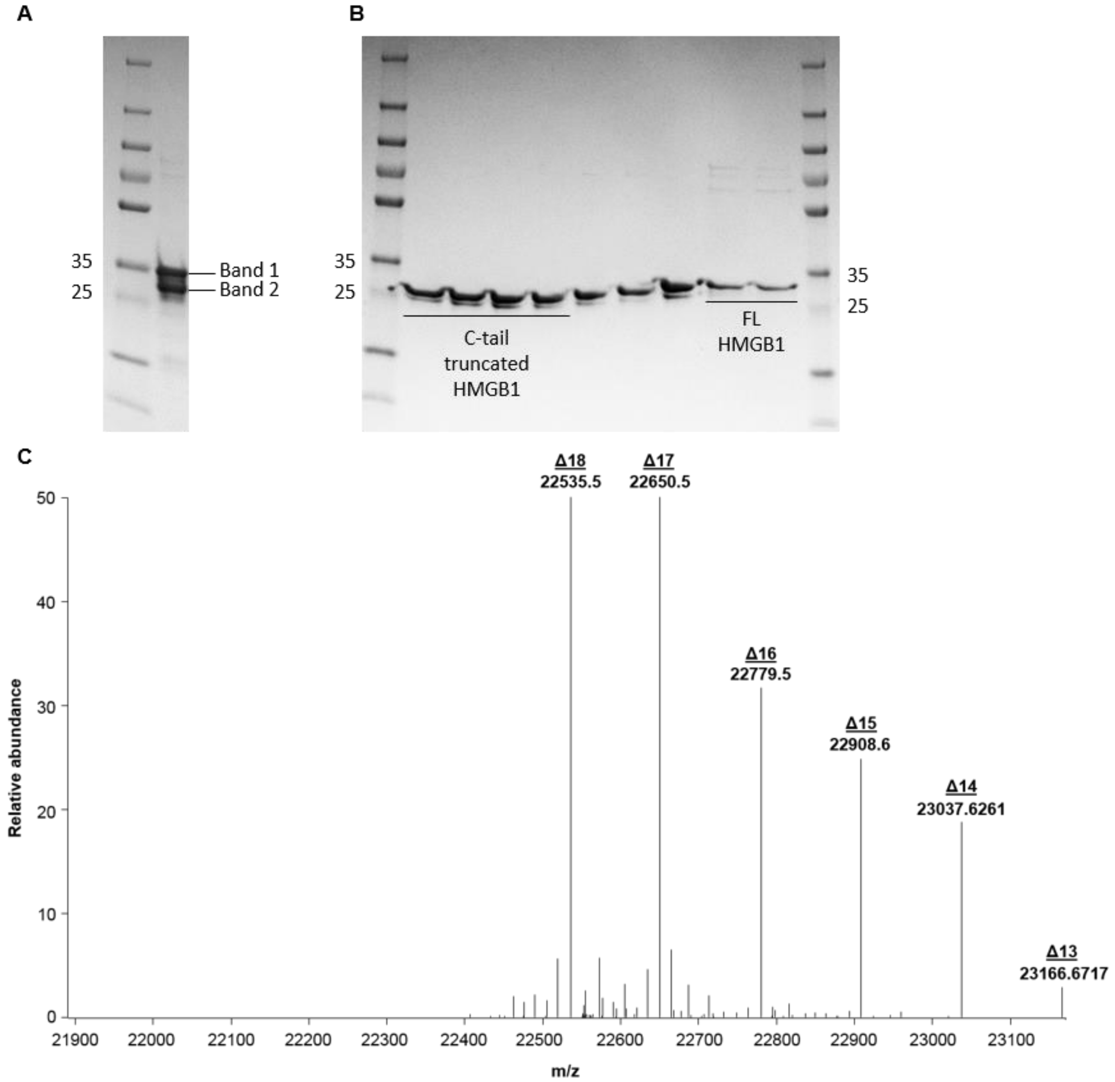
A
B
35
25
Band 1
Band 2
35
25
35
25
C-tail truncated HMGB1
FL HMGB1
C
Δ18 22535.5
Δ17 22650.5
Δ16 22779.5
Δ15 22908.6
Δ14 23037.6261
Δ13 23166.6717
Relative abundance
0
10
20
30
40
50
21900
22000
22100
22200
22300
22400
22500
22600
22700
22800
22900
23000
23100
m/z

Fig S2.

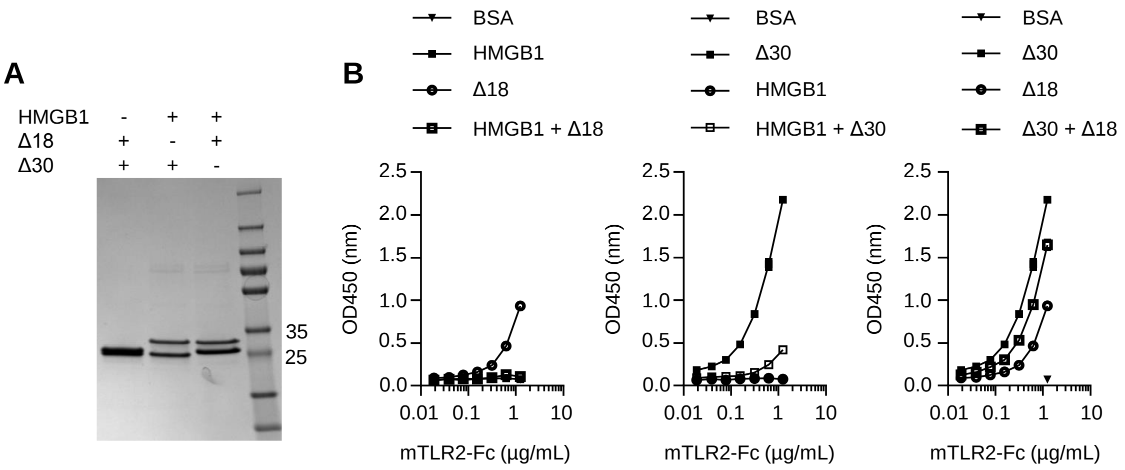
A
HMGB1 - + +
Δ18 + - +
Δ30 + + -
35
25
B
BSA
HMGB1
Δ18
HMGB1 + Δ18
BSA
Δ30
HMGB1
HMGB1 + Δ30
BSA
Δ30
Δ18
Δ30 + Δ18
OD450 (nm)
2.5
2.0
1.5
1.0
0.5
0.0
0.01
0.1
1
10
mTLR2-Fc (µg/mL)

48 **Fig S3.**

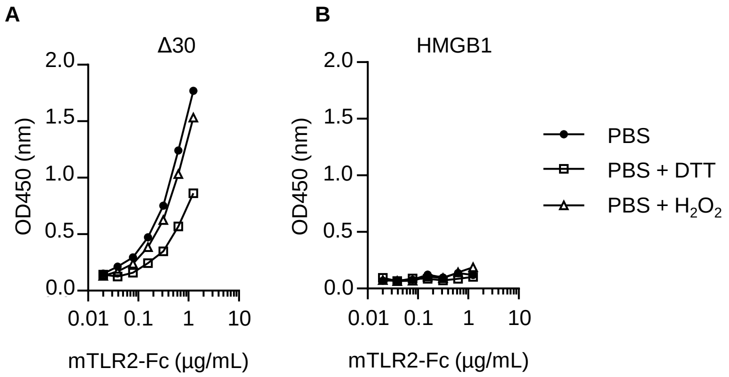

A
Δ30
2.0
1.5
1.0
0.5
0.0
OD450 (nm)
0.01
0.1
1
10
mTLR2-Fc (µg/mL)
B
HMGB1
2.0
1.5
1.0
0.5
0.0
OD450 (nm)
0.01
0.1
1
10
mTLR2-Fc (µg/mL)
PBS
PBS + DTT
PBS + H_2O_2
